# The Launch of the European Patients' Academy on Therapeutic Innovation in the Netherlands: A Qualitative Multi-Stakeholder Analysis

**DOI:** 10.3389/fmed.2020.00558

**Published:** 2020-09-11

**Authors:** Annemiek van Rensen, Helene R Voogdt-Pruis, Eva Vroonland

**Affiliations:** ^1^PGOsupport, Utrecht, Netherlands; ^2^Department of Global Health, Julius Center for Health Sciences and Primary Care, University Medical Center Utrecht, Utrecht, Netherlands

**Keywords:** patient involvement, drug development, medicines research, EUPATI, patient engagement, training, patient representatives

## Abstract

**Background:** Involving patients' representatives in the research and development of medicinal products (medicines R&D) leads to better medical treatment. In 2014, the European Patients' Academy on Therapeutic Innovation (EUPATI) was started with the goal of increasing the capacity and capabilities of patient representatives in this field. To make this academy more accessible and applicable for the Netherlands, a Dutch version was launched in September 2019. To explore the options for a durable infrastructure for organizing the Dutch EUPATI course, a multi-stakeholder qualitative study was done. The views of various stakeholders from pharmaceutical industry, governmental organizations, patient organizations, and the academic world were examined about the benefits and challenges of this course for patient involvement in medicines R&D.

**Methods:** From April to June 2019, 10 semi-structured interviews were completed, each with two representatives of all stakeholders involved. In addition, individual Dutch graduates of the European EUPATI (EUPATI fellows) were consulted *via* an e-mail questionnaire. Using a directed content analysis based on the Business Canvas Model, the transcribed interviews were coded, analyzed, and final attributes consolidated.

**Results:** The semi-structured interviews and completed questionnaires explored how the stakeholders are aiming to assist patient involvement in medicines R&D through the Dutch EUPATI course. The building blocks of the Business Canvas Model were described with concrete attributes for making the business case. Stakeholders stated that the Dutch EUPATI course was an incentive for patient involvement in medicines development, for patient-oriented research and outcomes, for the availability of patient representatives (expert ones in particular), and for the content and representation quality of patient representatives. The key values for collaborating in the network as mentioned by the stakeholders were neutrality, patients' interests, equality, independence, shared objectives, long-term commitment, transparency, understanding, trust, and respect.

**Conclusions:** Patient involvement in medicines R&D is evolving and the demand for qualified patient representatives is growing. Dutch stakeholders confirmed the added value of the patients' academy and expressed their willingness to contribute. Important values and conditions for long term collaboration were formulated.

## Introduction

There is increasing awareness that involvement of patient representatives in medicines research and development (R&D) benefits the development process of medicinal products (1–3). Integrating patients' priorities, perspectives, and knowledge throughout the medicines R&D process may allow better treatments with more relevant outcomes to become available for patients and their healthcare providers with shorter development timelines (4). To increase the capacity and capabilities of patient representatives in understanding and contributing to medicines R&D, the European Patients' Academy on Therapeutic Innovation (EUPATI) patient expert training course for patient representatives was launched in 2014. This EUPATI training course takes 14 months and comprises six online modules and several face-to-face meetings. Topics addressed are discovery and development of medicinal products, non-clinical development, clinical development, registration, safety, pharmacovigilance, and health technology assessment (5). EUPATI started as a project of the Innovative Medicines Initiative Joint Undertaking and in 2017 it continued as a pan-European public-private partnership program of the European Patients' Forum^1^ Since 2014, EUPATI has trained around 150 patient representatives (hereinafter “EUPATI fellows”) from many European countries (6), including eight patient representatives from the Netherlands. Due to the high demand for expert patients, the potential language barrier of the course being in English and the need to educate representatives not only about the European but also about the Dutch context (such as specific Dutch regulations, institutions, and materials), the Dutch EUPATI fellows pursued the idea of a Dutch version of the course. They therefore approached PGOsupport, a Dutch not-for-profit network organization that helps patient organizations and their counterparts accelerate patient involvement initiatives. In 2018, PGOsupport formed a multi-stakeholder steering group with representatives from the pharmaceutical industry, governmental organizations, patient organizations, and the academic world to initiate preparatory activities for the Dutch version of the EUPATI course. As recommended by the steering group, working groups were formed for each course module to adapt the online modules of EUPATI to the Dutch context. Each working group included the expertise and perspective of patient representatives (mainly EUPATI fellows), government, the academic world, and industry. In 2019, all the online modules of the EUPATI course were adapted for the Dutch context. A durable Dutch EUPATI course will need an effective infrastructure within the network it is to be embedded in. In parallel with the development of the Dutch version of the EUPATI course, we set out to determine the optimal conditions for such an infrastructure in order to promote and establish a long-term partnership. A multi-stakeholder qualitative analysis was conducted to that end among all the stakeholders (pharmaceutical industry, governmental organizations, patients, and the academic world). In addition, the results of this analysis will be used for further roll-out of the Dutch version of EUPATI in the Netherlands and the agreements on collaboration between involved stakeholders that arise from it.

## Methods

### Aim

This multi-stakeholder qualitative study aimed to explore the business case for the Dutch version of the EUPATI course. Specific research questions were what attributes do stakeholders' representatives assign to the Dutch business case for EUPATI and what values they regard as important for collaboration among stakeholders.

### Study Design

From April to June 2019, before the official start of the first cohort of the EUPATI course in the Netherlands, a multi-stakeholder qualitative analysis was held among stakeholders involved, using semi-structured interviews and questionnaires.

### Study Population

All stakeholders from the pharmaceutical industry, governmental organizations, patient organizations, and the academic world who participated in the steering group and working groups were approached for a semi-structured face-to-face interview with two interviewers (HV, SvD). Per stakeholder, two representatives were present during the interview. In addition, all eight Dutch EUPATI fellows received a questionnaire *via* e-mail to let them add their personal views. Participants in the study thereby represented the perspectives of the patients, governmental bodies, the academic world, and industry. All participants were based in the Netherlands and familiar with the Dutch medicine R&D context. Table 1 provides an overview of study population: the organizations involved in the interviews and the mission of the organizations.

**Table 1:** Organizations involved in the interviews and the mission of the organizations.

**Type of stakeholder**	**Name**	**Mission**
Patient representatives	1 The Dutch Patient Federation (PF)	Umbrella organization representing around 200 patient and consumer organizations
	2 EUPATI fellows	−8 Dutch patient advocates, with experience on topics related to accessibility and quality of care: - representing a variety of patients groups, through a Dutch and/or international patient association - graduates of the European EUPATI training (cohorts 1-3) - no professional affiliation with medicine R&D
Governmental organizations	3 Netherlands Pharmacovigilance Centre (Lareb)	Analyzes the risks associated with medicines and the use of drugs and the Center of Expertise for adverse drug reactions
	4 Medicines Evaluation Board (CBG-MEB)	Regulates the quality, efficacy, and safety of medicines. National competent authority for medicine regulation
	5 National Health Care Institute (ZIN)	Ensures that healthcare is accessible, affordable, and good quality
	6 Ministry of Health, Welfare and Sport (VWS)	
Pharmaceutical industry	7 The Association Innovative Medicines (VIG)	Umbrella association for Dutch pharmaceutical companies; focus on R&D
	8 HollandBIO	Association that represents and connects the biotech industry sector
Academic world	9 University of Applied Sciences Utrecht	One lecturer from the pharmacy program participated
Multi-stakeholder organizations	10 Dutch Clinical Research Foundation (DCRF)	Foundation of representatives from umbrella organizations (academic medical centers, research organizations, pharmaceutical companies, medical ethics committees, and patient organizations). Facilitates clinical research
	11 Health~Holland	Public-private partnership between the pharmaceutical industry, academic world and government. For innovations in the health and life sciences industry
Others	12 Organisation for health research and development (ZonMw)	Mainly supported by the ministry of VWS and funder of health research and implementation of research
	13 PGOsupport	A not-for-profit network organization, funded by ministry of VWS. supports and advises patient organizations. Holds a patient academy for several topics related to patient involvement

### Interviews and Questionnaires: The Business Canvas Model

The respected Business Canvas Model (BCM) by Osterwalder and Pigneur (7) was used for exploring the business case. In our study, stakeholders' representatives assigned attributes to the nine building blocks with the four main pillars of BCM. The four main pillars of BCM were defined as (i) “Product,” indicating the Dutch version of the EUPATI course, (ii) “Customers,” indicating the patient representatives, (iii) “Infrastructure,” encompassing organization and network, and (iv) “Financial,” referring to the costs and revenues of the Dutch version of the EUPATI course. The four pillars of BCM consist of nine building blocks: Value propositions (Product) Customer segments, Channels, Customer Relations (Customer), Key Activities, Key Resources, Partner Network (Infrastructure), Cost Structure, Revenue Streams (Finances).

### Data Collection

During the interview, which lasted 45–60 min, participants answered open-ended questions which probed their understanding of the business case according to the building blocks and attributes of BCM (7) (see Additional File 1). To ensure rich data collection, the topic list of the interview was sent before the interview, allowing participants to discuss these topics within their organizations before the interview. The interviews were recorded electronically and transcribed. In addition, the eight Dutch EUPATI fellows received a short questionnaire. The interview guide and questionnaire were created by the two independent interviewers (HV, SvD) and by the first author (AvR). All participants gave their written consent to participate in this study.

### Analysis

A directed content analysis approach (8) was used during the iterative transcript coding process. The codebook followed the building block categories of BCM (7). The attributes within the building blocks were refined during the analysis process to reflect emerging findings. The final coding scheme included eight building blocks (two building blocks were merged). A dual-coder process was used: two independent interviewers/researchers (SvD, HV) coded the transcripts separately and coding discrepancies between the two researchers were discussed and reconciled. The final attributes were re-analyzed and consolidated by two of the authors (AvR, HV).

## Results

### Participants

Ten semi-structured interviews were held with two representatives (a member of the steering group and their manager) of each of the respective stakeholder organizations (nine face-to-face and one by phone). One stakeholder—the Ministry of Health, Welfare and Sport—did not participate as they regarded themselves as an indirect stakeholder, in the context of this study. Five EUPATI fellows (63%) filled out the e-mail questionnaire.

### Views on the “Product”: Added Value of the Dutch Version of EUPATI

The first pillar of the Business Canvas Model—the “Product”—defines what value the Dutch version of EUPATI offers to customers and stakeholders (7). Stakeholders stated five main attributes of the Dutch version of EUPATI (Table 2):

1. The Dutch EUPATI course helps create an incentive for patient involvement in medicines development and policy in the Netherlands. “*It is important to consult patients. For example, we need to know which outcomes are relevant: what do patients consider as relevant topics for research, what are the needs of patients? Patient representatives are therefore needed for experiential panels, for assessment of research projects”*.
2. The Dutch EUPATI course will increase the relevance of medicinal products for patients.
  “*In doing so, we could avoid the development of medicinal products that are not really interesting for patients. It is important to know patients' experiences with medicines: is it comfortable for patients to use them or do they experience it as an excessive burden? Do the medicinal products fit with patients' needs and preferences? Patient involvement is needed to accelerate and improve important solutions*.
3. The Dutch EUPATI course increases the capacity of expert patient representatives available for patient involvement in medicines R&D.
  *The process of medicines development is quite complex. Patient representatives who are trained in this topic and who have knowledge of the process of medicine development are better equipped to provide good advice. EUPATI makes sure that patient representation is as professional as possible and that more patient representatives are trained. Usually the same patient representatives—often with a medical background—are involved in working groups. small patient groups can be better represented too*.
4. The Dutch EUPATI course increases the quality of advice from patient representatives.
  *EUPATI increases the knowledge and skills needed for patient representatives to be good spokespeople for a patient group or patient organization in the various stages of medicines development*.
5. The Dutch EUPATI course increases the “soft skills” of patient representatives.
  *Trained patient representatives are better equipped and probably less intimidated by other professional experts in the world of medicinal products. That will let them represent the interests of patients more effectively. Patient representatives need to work together wisely with the pharmaceutical industry and soft skills (e.g., conversation skills) are needed in addition to knowledge. Patient representatives should be able to make sure that other parties see their interests and the interests of patients who cannot represent themselves*.

**Table 2:** Product and customer interface of the Dutch EUPATI course according to stakeholders.

**Pillars**	**Building block**	**Attribute**	**Quotes (compilation)**
Product	1. Value proposition	Incentive for patient participation in drug development and policy	It is important to consult patients. For example, we need to know which outcomes are relevant: what do patients consider as relevant subjects for research, what are the needs of patients? Patient representatives are therefore needed for experiential panels, for assessment of research projects.
		Increase of relevancy of pharmaceutical products for patients	In doing so, we could avoid the development of pharmaceutical products that are not really interesting to patients. It is important to know patients' experiences with medicines; is it comfortable for patients to use them or do they experience it as an excessive burden? Do the medicines fit with patients' needs and preferences? Patient participation is needed to accelerate and improve important solutions.
		Availability of patient representatives	The process of drug development is quite complex. Patient representatives who are trained in this topic and who have knowledge of the process of drug development are better equipped to provide good advice. EUPATI makes sure that patient representation is as professional as possible and that more patient representatives are trained. Usually, the same patient representatives—often with a medical background—are involved in working groups. Small patient groups can be better represented too.
		Proper expert advice	EUPATI increases the knowledge and skills needed for patient representatives to be a good spokesperson for a patient group or patient organization in the different stages of drug development.
		Soft skills (Decisive representation)	Trained patient representatives are better equipped and probably less intimidated by other professional experts in the world of pharmaceuticals. That lets them represent the interests of patients better. Patient representatives need to cooperate wisely with the pharmaceutical industry and soft skills—for example conversation techniques—are needed in addition to knowledge. Patient representatives should be able to make sure that other parties see their interests.
Customer interface	2. Target customer	All patient groups, also “rare diseases”	A variation in professionalism among patient representatives exists. We are in need of proper representation for different patient groups. EUPATI should be accessible for patient representatives of different patient groups. Not only somatic disorders. Some patient representatives are dealing with sudden disease progression. That makes it sometimes hard to stay involved. More representatives of these patient groups should be trained probably.
	3. Distribution channel	To promote EUPATI and patient participation	As stakeholders, we would like to contribute to EUPATI by paying attention to it and to patient participation in drug development in our newsletters, *via* social media.
		Recruitment of students	It is important to connect the patient organizations both directly and *via* the umbrella patient organization.
	4. Relationship	(See block 7)	(See 7, Partner network).

In particular, the growing interest and willingness of both patient organizations and other stakeholders to actively engage in the medicine R&D process requires more patient representatives with knowledge and skills across all aspects/phases throughout the medicine R&D lifecycle, from pre-approval to post-marketing activities.

### Views on the “Customer”: Patient Representatives and Students

The second pillar (“Customer interface”) determines who the target customers are, how services are delivered and how relationships with customers are built (7). According to the stakeholders, the Dutch version of EUPATI should target patient representatives from all types of patient organizations, including rare diseases and psychiatric disorders (Table 2). Although the EUPATI course in itself may require a certain entry level of prior education, EUPATI students should learn how to represent the needs of patients with lower health literacy and/or intellectual capacities. For effective roll-out of the new Dutch EUPATI course and to ensure continuity of the engagement processes, it is important to reach as many patient organizations as possible. All the stakeholders expressed their willingness to contribute to promoting EUPATI within their networks (the “Distribution channel” building block), to recruit future EUPATI students and to increase the call for expert patient involvement in the process of medicines R&D.

### Views on “Infrastructure Management”: Activities and Network

“Infrastructure management” is related to the activities, logistics, resources, and partnerships needed for building a sustainable Dutch EUPATI course (7). Stakeholders put forward several ideas about the activities and network of stakeholders and graduates, as well as the values they considered to be important in a partner network (Table 3. Attributes for the three building blocks Value Configuration, Core competencies, and Partner network).

1. Follow-up training and meetings: As additional activities to the regular EUPATI course, stakeholders advised organizing follow-up training and/or meetings for graduates in order to exchange experiences, letting them learn from each other and/or do training on the job. New students could meet graduates and learn from them as well (the “Value configuration” building block).
2. Update/expand the course: Stakeholders can contribute by adding their “core competencies” to the EUPATI course: “*In the coming years, new treatments, therapeutic innovations, and policies will emerge. As stakeholders, we could help keep the content of EUPATI up to date.”* Furthermore, they could assist by making an active contribution to on-site training programs for specific skills related to effective patient involvement.
3. Partner network: Stakeholders see opportunities for mutual agenda setting: “*This network could address important topics on medicines R&D as a collective, as well as exchanging details and keeping each other informed about everyone's activities.”* Graduates of the European and national programs should stay in contact, preferably as a strong EUPATI alumni network across Europe. A visible network of stakeholders and patient representatives will lower the threshold for a broad range of patient involvement initiatives.

**Table 3:** Infrastructure management and financial aspects of the Dutch EUPATI course according to stakeholders.

**Pillars**	**Building block**	**Attribute**	**Quotes (compilation)**
Infrastructure management	5. Value configuration	Follow-up training and meetings	It is advisable to organize follow-up training and/or meetings for graduates, in order to exchange experiences, to learn from each other; to train on the job. New students could meet graduates and learn from them as well.
	6. Core competencies	Regular update: new treatment/procedures	In the coming years, new treatment, therapies, and policy will appear. As stakeholders, we could help keep the content of EUPATI up to date.
		Educational	In relation to certain skills—for example negotiating, conversation techniques, or educational/didactic methods.
	7. Partner network	Network of stakeholders	This network could address important topics on drug development together, exchanging details, and keeping each other informed of their activities.
		Network of graduated patient representatives	Fellows and graduates should stay in contact with each other and build a strong network. Also with EUPATI Europe. The network can be accessed easily for organizations in cases where patient involvement is desired. It is also important that patient representatives stay in close contact with their own patient organization.
		Important values 1 Neutrality 2 Patients' interests 3 Equality 4 Independence 5 Shared objective 6 Long-term commitment 7 Transparency 8 Understanding 9 Trust 10 Respect	PGOsupport—an organization for training and supporting patient organizations and patient representatives in the Netherlands—is the designated organization for leading EUPATI NL (neutrality, patients' interest). Cooperation in the network of stakeholders should be based on “equality and independence.” All stakeholders do have a specific contribution to and role in the process of drug development. While some individual interests could oppose each other—for example those of the pharmaceutical industry and other stakeholders—stakeholders should make their interests in cooperation transparent immediately and discuss how to deal with potential differences, with an openness or willingness to understand divergent values. All stakeholders are ethically obliged to cooperate in order to improve the value of medicines for patients. Although there are some reservations on working with the pharmaceutical industry, stakeholders should all work together, with mutual trust and respect. A public private partnership needs to be built with the pharmaceutical industry and other stakeholders—with a well-thought governance structure. The network should work together practically, with a small steering group who have “lean” meetings with each other. Working on long-term commitment is an important prerequisite for continued cooperation in the network, with transparency about financing flows.
		Conditions	1. Avoid the image of being a “mouthpiece” of the pharmaceutical industry, avoid any kind of advertising 2. Only umbrella organizations of pharmaceutical companies should be involved in the steering group 3. Work together according to the official code of conduct for organizations on cooperation with the pharmaceutical industry. Make explicit agreements on the conditions.
Financial aspects	8. Cost structure	Financial contribution	Stakeholders cannot provide long-term financial support. Some gave room for financial support for a certain period of time. One of these—a policy organization—promised support if the umbrella organization of pharmaceutical industries would do the same. Financial support should be collected in an independent depot.
		In-kind contribution	All stakeholders would like to contribute to EUPATI by providing services and materials, for example by providing experts for know-how or presentations or by use of locations for meetings.
	9. Revenue model	Evaluation of EUPATI	An evaluation study should give a clear picture of the benefits of EUPATI for students and stakeholders. How did graduates participate in drug development? In addition, the evaluation study provides suggestions for improvement: for example, is the content relevant and up-to-date?.
		Diffusion of knowledge	Graduates could spread the knowledge about patient participation in drug development to their patient organizations.

Concerning the organizational structure of the network around EUPATI: the stakeholders agreed that PGOsupport—an independent foundation with an established training facility for patient representatives in the Netherlands since 2008—would be the designated organization to lead the Dutch EUPATI course. In that way, neutrality, patient-centeredness, and independence, as well as optimal utilization of existing knowledge about patient expert training, can be guaranteed. Furthermore, cooperation in the network of stakeholders should be based on “equality.” This is despite the fact that “*all stakeholders do have a specific contribution to and role in the process of medicine development*,” and should therefore contribute from their specific backgrounds and expertise. Working on long-term commitment is an important prerequisite for long-term cooperation, with transparency about financial flows. Values considered by the stakeholders to be important for the collaboration in the partner network were neutrality, patient-centeredness, equality, independence, transparency, understanding, trust, respect, and having a shared objective with long-term commitment. Finally, stakeholders recommended that the network should find a “lean” and efficient way of working—preferably with a small steering group, representative of the main stakeholder groups and within existing national and other structures.

The stakeholders addressed the various challenges for the network. One of the perceived challenges concerned transparency and openness of individual interests: “*While some interests could oppose each other—for example those of the pharmaceutical industry vs. other stakeholders—stakeholders should explain their motives for joining the EUPATI network immediately and discuss how to deal with potential differences, with an open mind and willingness to understand divergent values*.” In addition, due to the dense network of patient organizations in the Netherlands, one stakeholder warned against an “exclusive” and isolated network of expert patients and urged aiming for close relationships with the existing network of patient organizations. Finally, although some reservations about close relationships with the pharmaceutical industry were voiced, the stakeholders unanimously identified the industry as an essential partner for the Dutch EUPATI course. A public private partnership will need to be built by all stakeholders based on mutual trust and respect, with a well-thought through governance structure. The stakeholders identified the following conditions for collaboration with the pharmaceutical industry that should be made explicit in a written agreement: (1). any suggestion of being a “mouthpiece” of the pharmaceutical industry should be avoided; (2). only umbrella organizations of pharmaceutical companies should be involved in the steering group; (3). Existing codes of conducts should be followed when designing the public private partnership structure.

### Views on the “Financial Aspects”: Cost and Revenue

The estimated cost for one cohort of the Dutch version of EUPATI is around 150 k euros, consisting of personnel costs for teaching at the face-to-face meetings, personal coaching of students during the online course and maintenance of the online course environment (“virtual classroom”). Annual costs may vary depending on in-kind contributions from individual stakeholders. As the current policy of the European EUPATI program is to deliver these services and the education program without subscription fees for patient representatives (students) and their organizations, the stakeholders have been asked for a financial and/or in-kind contribution to the Dutch EUPATI course. Unfortunately, long-term financial support could not be guaranteed upfront (Table 3 Attributes for building blocks Cost structure and Revenue model). Some parties saw openings for financial support for a certain period of time. One of these—a governmental organization—suggested a contribution as co-financer, provided the industrial partner would do the same. As a general prerequisite, the financial contributions should be distributed from an independent depot, with no ties to the individual sponsor. Concerning in-kind contributions, all stakeholders expressed an unconditional intention to provide expertise (e.g., as a guest lecturer), facilities (e.g., for meetings) and materials. As the “Revenue model,” the stakeholders recommended an independent periodic evaluation study of EUPATI in order to identify benefits and added value of EUPATI for students and stakeholders. How did graduates engage in medicine development activities? What was the impact? In addition, the evaluation study will provide suggestions for improving the Dutch EUPATI course: for example, is the content relevant and up to date? Are additional course modules relevant for the Dutch context? Another benefit of EUPATI is the diffusion of knowledge about patient involvement in medicine R&D. Both graduates and involved stakeholders will act as an ambassador for (knowledge on) patient involvement in drug development to members of their respective networks.

## Conclusion

Patient involvement in medicine R&D is evolving and demand for qualified patient representatives is growing. Dutch stakeholders confirmed the added value of the existing EUPATI patients' academy and expressed their willingness to contribute to a durable EUPATI program in the Netherlands. Important values and conditions were formulated.

## Discussion

A multi-stakeholder qualitative analysis was conducted to explore the values and visions of stakeholders from the academic, governmental, industrial, and patient perspectives for a sustainable EUPATI program in the Netherlands. Building blocks within the BCM were used to structure both data collection and analysis.

### Dutch EUPATI Course Adds Value to Medicines R and D Ecosystem

Although the main purpose of our study was not to investigate the added value of patient involvement in medicines R&D as such, our respondent outcomes overlap with earlier findings from others in this regard (9). In general, stakeholders share a sense of urgency and express an overall interest with respect to patient involvement in medicines R&D. They furthermore share a strong wish for capacity and capability building and agree that a Dutch version of the EUPATI training program could meet the needs that arise from such a wish. Stakeholders expect EUPATI to be a relevant and high-quality training program and thereby to contribute to an increase in the pool of patient experts available for patient involvement activities. It is believed that in addition to their valuable experiences as a patient, substantial knowledge of terminology, and the complex process of development of medicinal products is necessary for patients to be equal partners in the dialogue with stakeholders (9–11). By focusing on both technical knowledge and relevant skills in general, not only will the quality of the outcomes of the engagement activity benefit (e.g., the patient's advice), but so will the engagement process as such. Klingmann et al. (12) have already stated that if patients can obtain knowledge through education, multiple impactful roles in the medicine development process come within reach. Training may also be beneficial for restoring the power balance between patients and professionals from other stakeholder groups. According to Abma (13), patients need to develop a strong, self-conscious position before they are ready to participate and enter into a dialogue with researchers about their agenda. As well as concrete output from trained patient representatives, as graduates of a particular cohort, stakeholders also believe that a Dutch EUPATI course will indirectly add to the incentive for patient involvement in the Netherlands in general. As such, this is in line with previous observations that a combined effort of governmental organizations, the academic world, patient organizations, health foundations, and the pharmaceutical industry to share knowledge and resources in order to address the challenges of pharmaceutical innovation will be beneficial for all (14). Internationally, the PARADIGM consortium is currently investigating the return on investment of patient involvement initiatives and metrics for quantitative and qualitative assessment. The initial publications from this consortium suggest a key role for education and empowerment in evaluating benefits and costs (15).

### High-Level Training for Patient Representatives

Stakeholders believe the Dutch EUPATI course should target a broad population of patient representatives, including somatic, psychiatric, and rare conditions. By doing so, it may complement the pre-existing, more disease-specific coaching and training programs that various patient organizations provide for their patient advocates. Dedicated and high-level training programs with contributions from relevant stakeholder parties have until now been exclusively directed at professionals.^2^ The Dutch EUPATI course means that a “generic” and high-level training program on this topic, established in collaboration with and directed at patient representatives, will become available in the Netherlands for the first time.

### Benefits Extend the Aspect of Education

Although the primary deliverable of the Dutch EUPATI network is an educational program, stakeholders have identified other opportunities, aims and benefits for the collaboration as well. Mutual agenda setting and knowledge exchange were identified by some. A brief inventory of some of the existing EUPATI National Platforms (ENPs)^3^ in other countries reveals a diverse picture of approaches in different countries. Whereas, training is the central focus for most ENPs, network building, and raising awareness appear to be important as well (16). As an additional spin-off, active participation by all stakeholders within the Dutch EUPATI context is expected to lower thresholds for future contacts between stakeholders and patient representatives. Face-to-face contacts during expert group meetings and on-site training elements will add to the aspect of “getting to know your future discussion partner already at an early stage”. This is expected to establish mutual trust and understanding for the longer term (17, 18).

### Important Values for a EUPATI Network and Training Program in the Netherlands

Stakeholders identified important key values and principles at an infrastructural level when asked to express their worries and expectations for their collaboration in a Dutch EUPATI network. Transparency, equality, trust, independence, and respect are among the most frequently mentioned values. Furthermore, alignment of goals was seen as essential for a successful inter-organizational collaboration, as has already been described by others (18). The values and key principles that were derived from our multi-stakeholder analysis overlap nicely with international concepts as laid down in various codes of conduct and frameworks from regulatory authorities [such as EMA (1)], the industry [such as EFPIA (19)], and patient organizations [such as Eurordis (20)] that describe the guiding principles of patient involvement between two or more stakeholders. International programs for advancing patient involvement in medicines R&D, such as the Patient-Focused Medicine Development initiative^4^, furthermore draw upon these values and principles by providing tools and resources for all stakeholder parties. Although national specifics will apply, these findings let us employ available resources by anticipating that an effective and durable Dutch EUPATI infrastructure could be mapped according to the international example.

### Collaboration With the Pharmaceutical Industry: Valuable and Delicate

The position of the industrial partner in the Dutch EUPATI network deserves further consideration. All stakeholders regard the pharmaceutical industry as both a relevant and an essential partner for the Dutch EUPATI course. Combining knowledge and resources in order to address the challenges of pharmaceutical innovation has been proved to be beneficial for government organizations, the academic world, patient organizations, health foundations, and the pharmaceutical industry, as they all share that interest (14). As involvement of an industrial partner is prone to be affected by a negative public image (21, 22), careful deliberation is crucial to avoid a negative impact on the credibility of the training program. Combining the recommendations of several stakeholders in this regard, together with the available international and other guidelines on collaboration with the industry (5) will let us establish an internationally accepted modus operandi, customized for the Dutch situation. Trust, alignment of goals, balancing power, and open communication are among the underlying concepts to be considered (9, 17, 18).

### Strengths and Limitations of the Research

The strength of this study is the objective, qualitative in-depth analysis among multiple stakeholders involved in medicines R&D in the Netherlands. It let us combine the results from the governmental, academic, patient and pharmaceutical perspectives, and hence draw conclusions about collaboration between multiple stakeholders to promote and establish patient involvement in medicines R&D by means of a relevant and high-level educational program, focused on patient representatives. The in-depth interviews in this study were held by two independent interviewers who did not come from any of the stakeholders. Furthermore, the business canvas model was used to compile the topic list, ensuring that all important attributes for building a sustainable Academy have been taken into consideration. This study, however, also has some weaknesses. Because participation in our study was partly selective—participants were already part of the steering group—participants were more likely to be motivated toward patient involvement, rendering our study population probably not representative of all the stakeholders involved in medicines R&D. As a next step, we would recommend to also involve principal investigators responsible for clinical trial execution, for example in a hospital, to explore specific expectations and needs for this group as well. Although we used the business canvas model to compile the topic list, it is possible we missed topics that would have been identified if a different model had been used. Furthermore, results about the collaboration between stakeholders are tied to a single point in time, so important information could have been missed in understanding the long-term sustainability of collaborations. Continued monitoring the actual collaboration between EUPATI students, graduates, and other stakeholders and research into this is recommended.

## Data Availability Statement

The raw data supporting the conclusions of this article will be made available by the authors, without undue reservation.

## Ethics Statement

Ethical review and approval was not required for the study on human participants in accordance with the local legislation and institutional requirements. Written informed consent to participate in this study was provided by the participants.

## Author Contributions

AR, EV, and HV-P: academy design and drafted the work or substantively revised it. HV-P and AR: study design, data analysis, and data interpretation. HV-P: data acquisition. All authors read and approved the final manuscript.

## Conflict of Interest

The authors declare that the research was conducted in the absence of any commercial or financial relationships that could be construed as a potential conflict of interest.

## Footnotes

^1^https://www.eupati.eu

^2^For example: https://www.pauljanssenfuturelab.eu/ or https://www.sir-era.eu/.

^3^https://www.eupati.eu/#eupcrib

^4^https://patientfocusedmedicine.org/

## References

[1] European Medicines Agency. Revised Framework for Interaction Between the European Medicines Agency and Patients and Consumers and Their Organisations. London: EMA/637573/2014 (2014).30175100

[2] Hoos A Anderson J Boutin M Geissler J Johnston G Joos A. Partnering with patients in the development and lifecycle of medicines: a call for action. Therap Innovat Regul Sci. (2015) 49:929–39. 10.1177/2168479015580384 26539338 PMC4616907

[3] EPF European Commission Proposal for a Regulation on Clinical Trials. (2012). (COM369 final) Position Statement (Bruxelles).

[4] Warner K See W Haerry D Klingmann I Hunter A May M EUPATI guidance for patient involvement in medicines research development (R&D); guidance for pharmaceutical industry-led medicines R&D. Front Med. 5:270 10.3389/fmed.2018.00270 PMC6190844 30356834

[5] Geissler J Ryll B Leto S Uhlenhopp M. Improving patient involvement in medicines research and development: a practical roadmap. Therap Innovat Regul Sci. (2017) 51:216847901770640. 10.1177/2168479017706405 30231692

[6] Spindler P Lima BS. Editorial: The European Patients Academy on Therapeutic Innovation (EUPATI) guidelines on patient involvement in research and development. Front Med. (2018) 5:310. 10.3389/fmed.2018.00310 30467543 PMC6236003

[7] Osterwalder A Pigneur Y Tucci CL Clarifying business models: origins, present, and future of the concept. Commun Assoc Information Syst. (2005) 16:1–25. 10.17705/1CAIS.01601

[8] Hsieh HF Shannon SE. Three approaches to qualitative content analysis. Qual Health Res. (2005) 15:1277–88. 10.1177/1049732305276687 16204405

[9] Hansen MB Norgaard LS Hallgreen CE How and why to involve patients in drug development: perspectives from the pharmaceutical industry, regulatory authorities, patient organizations. Therap Innov Regul Sci. (2020) 25:577–85. 10.1177/2168479019864294 33301145

[10] Chakradhar S. Training on trials: patients taught the language of drug development. Nat Med. (2015) 21:209–10. 10.1038/nm0315-209 25742451

[11] Wicks P Richards T Denegri S Godlee F. Patients roles and rights in research. BMJ. (2018) 362:k3193. 10.1136/bmj.k3193 30045909

[12] Klingmann I Heckenberg A Warner K Haerry D Hunter A May M. EUPATI and patients in medicines research and development: guidance for patient involvement in ethical review of clinical trials. Front Med. (2018) 5:251. 10.3389/fmed.2018.00251 30246010 PMC6137130

[13] Abma TA Dialogue and deliberation: new approaches to including patients in setting health and healthcare research agendas. Action Res. (2019) 17:429–50. 10.1177/1476750318757850

[14] Reypens C Lievens A Blazevic V Leveraging value in multi- stakeholder innovation networks: a process framework for value co-creation and capture. Indust Market Manag. (2014) 56:40–50. 10.1016/j.indmarman.2016.03.005

[15] Vat LE Finlay T Schuitmaker-Warnaar TJ Fahy N Robinson P Boudes M. Evaluating the “return on patient engagement initiatives” in medicines research and development: a literature review. Health Expect. (2020) 23:5–18. 10.1111/hex.12951 31489988 PMC6978865

[16] Grine L Janssens R van Overbeeke E Derijcke D Silva M Delys B. Improving patient involvement in the lifecycle of medicines: insights from the EUPATI BE survey. Front Med. (2020) 7:36. 10.3389/fmed.2020.00036 32118020 PMC7031274

[17] Dey RM de Vries MJ Bosnic-Anticevich S. Collaboration in chronic care: unpacking the relationship of pharmacists and general medical practitioners in primary care. Int J Pharm Pract. (2011) 19:21–9. 10.1111/j.2042-7174.2010.00070.x 21235656

[18] D'Amour D Ferrada-Videla M San Martin Rodriguez L Beaulieu MD. The conceptual basis for interprofessional collaboration: core concepts and theoretical frameworks. J Interprof Care. (2005) 19(Suppl. 1):116–31. 10.1080/13561820500082529 16096150

[19] European Federation of Pharmaceutical Industries and Associations Efpia Code of Practice on Relationships Between the Pharmaceutical Industry and Patient Organisations. (2011). Available online at: https://www.efpia.eu/media/24310/3c_efpia-code-of-practice-on-relationships-pharmapluspt-orgs.pdf (accessed July 2, 2020).

[20] European organisation for rare diseases Code of Practice Between Patients' Organisations and the Healthcare Industry. Available online at: https://www.eurordis.org/sites/default/files/thumbnails/0904-PO-Code%20of%20practice.pdf (accessed July 2, 2020).

[21] Parssons S Starling B Mullan-Jensen C Tham S Warner K Wever K. What do pharmaceutical industry professionals in Europe believe about involving patients and the public in research and development of medicines? A qualitative interview study. BMJ Open. (2016) 6:e008928. 10.1136/bmjopen-2015-008928 26743701 PMC4716253

[22] Light Lexchin DW JR. Pharmaceutical research and development: what do we get for all that money? BMJ. (2012) 244:e4348. 10.2139/ssrn.2262843 22872694

