# Supplementary material for: The Launch of the European Patients' Academy on Therapeutic Innovation in the Netherlands: A Qualitative Multi-Stakeholder Analysis
Source: Front Med (Lausanne). 2020 Sep 11;7:558. doi: 10.3389/fmed.2020.00558 (PMC7533595; doi:10.3389/fmed.2020.00558)
Supplement: Supplementary file 1 [file Table_1.docx]

The launch of the European Patients’ Academy on Therapeutic Innovation in the Netherlands: A Qualitative Multi-stakeholder Analysis

Annemiek van Rensen^1^, Helene R Voogdt-Pruis^2^, Eva Vroonland^1^

^1^ PGOsupport, Daltonlaan 600, 3584 BK, Utrecht, Netherlands

^2^University Medical Center Utrecht, Julius Center for Health Sciences and Primary Care, Department of Global Health, Utrecht, Netherlands

* Correspondence: Annemiek van Rensen, a.vanrensen@pgosupport.nl

# Supplementary Material

# Additional File 1. Interview Guide: Topic List

# *For stakeholders*

# Product: Value proposition: Added value of the Dutch EUPATI course (for their organization, stakeholders, the Netherlands, students, Dutch healthcare system, etc.). Solution to current problems/needs; Measuring added value; Possible negative side effects or risks.

# Customer interface: Target customer: specific target groups needed (therapeutic area, setting, educational level, etc.) and arguments (needs, finances, etc.)

# Customer interface: Distribution channel: promotion of the Dutch EUPATI course and patient involvement in medicine R&D, recruitment of students

# Customer interface: Relationships

# Infrastructure: Key partners: Network of stakeholders; Network of patient representatives who have graduated; Important values and conditions for collaboration; Position of PGOsupport;

# Financial aspects: Cost structure: financial and in-kind contributions

# Financial aspect: Revenue model: return on investment of the Dutch EUPATI course

# Infrastructure management: Resources needed

# Infrastructure management: Key activities for durable infrastructure

# *For graduates of the European EUPATI course (“EUPATI fellows”)*

# Product: Value proposition: Added value of the Dutch EUPATI course (for stakeholders, the Netherlands, students, Dutch healthcare system, etc.). Solution to current problems/needs; Measuring added value; Possible negative side effects or risks;

# Customer interface: Relationships

# Infrastructure: Key partners: Network of stakeholders; Network of patient representatives who have graduated; Important values and conditions for collaboration; Position of PGOsupport
